# Embedding Learning with Triple Trustiness on Noisy Knowledge Graph

**DOI:** 10.3390/e21111083

**Published:** 2019-11-06

**Authors:** Yu Zhao, Huali Feng, Patrick Gallinari

**Affiliations:** 1 Financial Intelligence and Financial Engineering Key Laboratory of Sichuan Province, School of Economic Information Engineering, Southwestern University of Finance and Economics, Chengdu 611130, China; 2 Laboratoire d’Informatique de Paris 6 (LIP6), Universit Pierre et Marie Curie, 75252 Paris, France; patrick.gallinari@lip6.fr

**Keywords:** knowledge graph, embedding learning, cross entropy, noise detection, triple trustiness

## Abstract

Embedding learning on knowledge graphs (KGs) aims to encode all entities and relationships into a continuous vector space, which provides an effective and flexible method to implement downstream knowledge-driven artificial intelligence (AI) and natural language processing (NLP) tasks. Since KG construction usually involves automatic mechanisms with less human supervision, it inevitably brings in plenty of noises to KGs. However, most conventional KG embedding approaches inappropriately assume that all facts in existing KGs are completely correct and ignore noise issues, which brings about potentially serious errors. To address this issue, in this paper we propose a novel approach to learn embeddings with **triple trustiness** on KGs, which takes possible noises into consideration. Specifically, we calculate the trustiness value of triples according to the rich and relatively reliable information from large amounts of entity type instances and entity descriptions in KGs. In addition, we present a cross-entropy based loss function for model optimization. In experiments, we evaluate our models on KG noise detection, KG completion and classification. Through extensive experiments on three datasets, we demonstrate that our proposed model can learn better embeddings than all baselines on noisy KGs.

I am convinced that the crux of the problem of learning is recognizing relationships and being able to use them. *Christopher Strachey in a letter to Alan Turing, 1954*

## 1. Introduction

Knowledge graphs (KGs) provide effective well-structured relational information between entities. A typical KG usually consists of a huge amount of knowledge triples in the form of (*head entity*, *relationship*, *tail entity*) (denoted (h,r,t)), e.g., (*Barack Obama, was_born_in, Hawaii*). KG embedding aims at learning embeddings of all entities and relationships, which usually are used to promote down-stream knowledge-driven artificial intelligence (AI) and natural language processing (NLP) tasks, such as human-like reasoning, semantic parsing [1], question answering [2,3], relation extraction [4,5], speech generation [6], etc.

The past decade has witnessed great surge in building web-scale KGs, such as Freebase [7], WordNet [8], YAGO [9], DBpedia [10], Google Knowledge Graph [11], and other domain-specific KGs. Recently, open information extraction (Open IE) [12], automatic neural relation extraction [13] and crowd-sourcing mechanism are widely used for KG construction, while these approaches inevitably bring ***noises*** in KG due to insufficient human supervision [14,15]. For instance, the recent open IE model on the benchmark achieves only 24% precision when the recall is 67% [16]. There are some existing approaches that have been proposed for knowledge graph embedding [17,18,19,20,21]. However, most conventional methods inappropriately assume that all facts in existing KGs are completely correct and ignore noise detection, which can lead to errors as the learning algorithm may treat incorrect facts as true ones. Hence, it is crucial to consider noises in knowledge graph embedding and down-stream tasks. As yet, the basic noise issue on knowledge graph embedding has not attracted enough attention [22]. Recently Xie et al. [23] propose to deal with noisy triples for representation learning. However, the calculation of its confidence value is not straightforward and needs too many intermediate processes with high complexity, especially for global path confidence. Moreover, they discard the external information which could provide rich information for judging triples.

**Figure 1:**
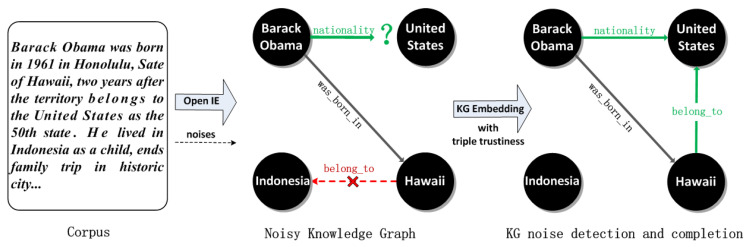
A brief illustration of our work. Knowledge graph faces the noise issue (×) after automatic construction via OpenIE. It’s expected to conduct knowledge graph (KG) embeddings learning with triple trustiness for noise detection. For instance, the noise *<Hawaii, belong_to, Indonesia>* is detected and updated to *<Hawaii, belong_to, United States>*. Moreover, our noisy KG embedding approach can be used to improve KG completion, such as the incomplete fact *<Barack Obama, nationality, ?>* is completed as a true fact *<Barack Obama, nationality, United States>* after noise correction, otherwise as a false one *<Barack Obama, nationality, Indonesia>* according to the noisy triple *<Hawaii, belong_to, Indonesia>*.

In this paper, we concentrate on learning embeddings on noisy knowledge graphs, which can deal with noises and embed all entries into low dimensional vector space. To address the noise issue, following the translation assumption [17], we propose a novel translating embedding learning approach with ***triple trustiness***, called **TransT**, which takes possible noises into consideration. The trustiness-based framework has been widely studied in the research field such as data mining [24]. Figure 1 demonstrates a brief illustration of our work. KG suffers from noises after automatic construction via OpenIE [12]. Such noises are expected to be considered and detected in learning embeddings with triple trustiness on KGs. For example, there exists a noise *<Hawaii, belong_to, Indonesia>* brought into the KG by OpenIE, which would be detected and ignored in embedding learning.

Specifically, TransT calculates the trustiness value of triples by considering two external auxiliary information: entity type instances and entity descriptions, which provide rich pragmatic and semantic information. Correspondingly, we build two novel sub-models for them. Moreover, we present a cross entropy based objective function for training all parameters of our model. We evaluate our model on three tasks including KG noise detection, KG completion, and triple classification. Experimental results demonstrate that our proposed model outperforms all baselines on all tasks, which confirms the capability of TransT in noisy KG embedding. The main work in this paper is concluded as follows:

- We propose a novel translating embedding model, TransT, for learning with triple trustiness on noisy knowledge graph by considering two external information, i.e., entity types and entity descriptions.
- Under this strategy, we propose two sub-models for calculating triple trustiness, one of which is estimated on newly generated entity type triples and another is measured with synthetic entity description triples.
- We present a cross entropy based approach for training model. The experimental results on three noisy datasets including *FB15K-N1, FB15K-N2* and *FB15K-N3* demonstrate the effectiveness of our proposed model.

The structure of the paper is as following. In Section 2, we will provide a brief review of related works. In Section 3, we describe the methodology of our model. In Section 4, we present cross entropy learning method. Section 5 presents experimental results followed by their discussion. Finally, Section 6 gives the conclusion and future directions of this research.

## 2. Related Work

### 2.1. Kg Noise Detection

There are various ways of building knowledge graphs, such as edited by crowdsourcing like Freebase [7], extracted from the large-scale semi-structured web like DBpedia [10], and open information extraction methods like Knowledge-Vault [11]. However, all of them inevitably suffer from noise interference due to insufficient human supervision when automatic mechanisms involve. Therefore, noise detection is essential and significant in knowledge automatic construction and knowledge-driven intelligent applications. Most knowledge graph noise detection works happen when constructing knowledge graphs [15,25,26]. These approaches are usually involved with huge human efforts, which are extremely labor-intensive and time-consuming. Recently, there are some works focusing on automatic KG noise detection [27]. Pellissier Tanon et al. [28] select features from contents, users, items [29], and P. et al. [30] propose to judge importance in graphs for nodes and edges. Paulheim and Bizer [31] propose the heuristic link-based type inference mechanism SDType, which can handle noisy and incorrect data. Melo and Paulheim [26] investigate the problem of error detection in relation assertions of knowledge graphs, and propose an error detection method which relies on path and type features used by a classifier for every relation in the graph exploiting local feature selection. Recently, Xie et al. [23] propose an embedding method (CKRL) with confidence to deal with noise detection, however, it ignores the rich semantic information in external nonstructural information which is strong evidence to judge triple quality. In this paper, we propose a knowledge graph embedding learning method with trustiness considering rich auxiliary information.

### 2.2. Knowledge Graph Embedding

In recent years knowledge graph embedding (see more in this survey [22]) has become a hot research topic. The key idea is to encode all the entities and relations in KG into a latent semantic vector space, so as to predict the probable truth of additional facts purely based on the existing triples in knowledge bases. Various embedding methods have been proposed in recent years. Bordes et al. [32] proposed a structured embedding model (SE), in which the basic idea was to transform the head entity and tail entity into a common latent space by the corresponding left and right projection matrices of the relation and then measured the similarity of the triple by $L_{1}$-norm distance in the embedding space. Bordes et al. [33] propose a semantic matching model (SME) for KGC. The main motivation of the model was that entities and predicate relations would share the same form of representation. It mapped all entities and predicate relations into a common latent space to delete the semantic difference between them. Socher et al. [19] propose a neural tensor network model (NTN), which tackled the issue of weak entity vector interaction through replacing a standard linear neural network layer with a bilinear tensor layer that directly relates left entity and right entity across multiple dimensions. The main intuition of the model was that each predicate relation would have different parts of semantic representation. Each slice of the predicate relation tensor was responsible for one class of entity pairs. Bordes et al. [17] propose a translating method (TransE) to model predicate relations by interpreting them as translations operating on the low-dimensional embedding of the left entity and right entity. Wang et al. [18] proposed a translating model (TransH), which builds predicate relation as a hyperplane with a translation operation on it. There are more models to conduct KG embedding, such as PIDE [34], RESCAL [35], TransG [21], TransR [36], TransD [37], Analogical [38], Convolutional2D [39], ProjE [40], ComplexE [20] and SSP [41], etc.

Moreover, the KG embedding approaches can be roughly classified into two categories according to the information they used: (1) those which learn embeddings only with KG at hand [17,18,36]; (2) those learning embeddings by combining existing KG with external heterogeneous information, e.g., entity hierarchical types [5,42], entity descriptions [41,43,44], plain text [45], and relation paths [46]. However, all these methods assume that all the facts in KG hold without noise, which is unreasonable especially for KGs constructed automatically without sufficient human supervision. In this paper, we concentrate on noisy KG embedding on the basis of the translation-based model (TransE), which is not difficult to be replaced with other enhanced KG embedding model [18,36].

### 2.3. Knowledge Graph Refinement

Knowledge graph refinement (KGR) is essential after automatic KG construction [11], since the result may never be perfect whichever approach is taken for constructing knowledge graph. Various methods for KGR have been proposed [25], which can differ along three distinct orthogonal dimensions: (i) the overall goal of the method, i.e., completion [17,19] vs. correction [15,26] of KG; (ii) the refinement target (e.g., relations between entities [19], entity types [47]), and (iii) the data used by the approach (i.e., only KG itself [17], or further external information [42,43]). However, most conventional approaches are only used for one goal as yet, while a combination between completion and error detection methods could be of great value [25]. Dong et al. [11] propose a joint approach with both prior knowledge stemmed from KG and external web content to estimate triple quality in KG construction, but lacking flexible ability in scale and reasoning capability without embedding strategy. Jia et al. [48] propose a crisscrossing neural network for KG completion and correction at the same time, while having high complexity and computational cost. In this paper, we introduce the triple trustiness for KGR, by considering the typical external heterogeneous source (i.e., entity type instances and entity descriptions) beyond the KG itself.

## 3. Methodology

**Notation.** For each triple (h,r,t), the head entity and tail entity h,t∈E and the relation r∈R, where E and R represent the sets of entities and relations respectively. D={(h,r,t)} stands for the overall training dataset with noises. $\tau_{h}$ and $\tau_{t}$ represent the hierarchical types of head and tail respectively. T represents the set of all types, $\tau_{h},\tau_{t}\in T$. $d_{h}$ and $d_{t}$ denote the descriptions of head and tail respectively. *w* represents the keyword in entity descriptions. W represents the set of keywords, w∈W.

To learn better embeddings on a noisy knowledge graph, we propose a concept triple trustiness for each triple fact. Triple trustiness denotes the evidential reliability of a triple which can be measured with the favor of external nonstructural auxiliary information.

### 3.1. Translating Embedding Model

To model entity triples, we first present a typical translating embedding model TransE [17], which was proposed to model relationships by interpreting them as translations operating on the low-dimensional embedding of the head entity and tail entity. The scoring function (the lower the better for correct triple) was as follows:

(1) $$M\left(h,r,t\right)={∥h+r-t∥}_{2}^{2}\;,$$

using $\ell_{2}$-norm, $h,r,t\in R^{\kappa }$.

### 3.2. Translating Embedding with Triple Trustiness

In order to detect noises and learn better embeddings with triple trustiness, we concentrate more on those triples with high evidential trustiness value. Following the translating assumption [17], we build the energy function E(·) of our translating embedding model with triple trustiness (TransT) as follows:

(2) $$E\left(D\right)=\sum_{\left(h,r,t\right)\in D}M\left(h,r,t\right)\cdot T\left(h,r,t\right).$$

**Figure 2:**
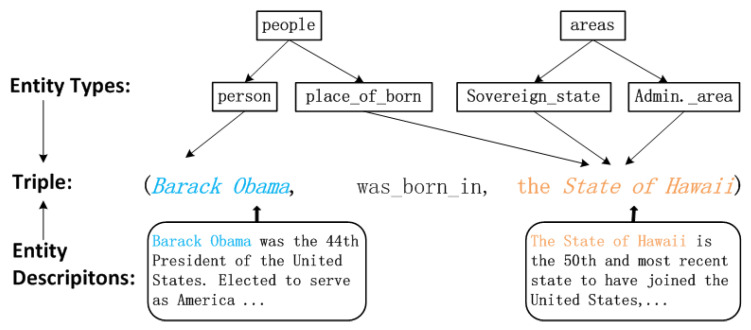
A triple *<Barack Obama, was_born_in, the State of Hawaii>* with its entity hierarchical types and entity descriptions. The left entity “*Barack Obama*” has a hierarchical type: “*/people/person*” and a description: “Barack Obama was the 44h President of the United States...”. The right entity “*the State of Hawaii*” possesses similar heterogeneous information.

The TransT energy function includes two parts. The first part M(h,r,t) represent the distance between head and tail with relation under translation assumption. A lower M(h,r,t) indicates that the embeddings of entities and relationships of this triple comply with the translation assumption well. We design the triple trustiness T(h,r,t) as the second part of our energy function. A fact with higher trustiness possesses higher quality, therefore, it should be more reasonably considered in learning embedding. Next we introduce two novel methods to measure triple trustiness according to external auxiliary sources, as in Figure 2.

### 3.3. Triple Trustiness

In this section, we introduce a novel method to measure triple trustiness with external nonstructural auxiliary information including entity types and entity descriptions.

#### 3.3.1. Triple Trustiness with Entity Types

We first utilize the entity hierarchical types for triple trustiness value estimation. Entity hierarchical types information implies different roles an entity may play in different scenarios [5]. Most typical knowledge graphs (e.g., Freebase [7], DBpedia [10]) have entity type information. Entity types usually consist of hierarchical structures, in which the lower granularity of semantic concepts is considered as the sub-type of entities. Generally, most entities possess more than one hierarchical type. For instance, in Figure 2, *the State of Hawaii* has a variety of types (e.g., */people/place_of_born, /areas/sovereign_state* and */areas/Administrative_area*) and shows different attributes under different types. The entity hierarchical types are strong evidence to estimate the triple trustiness. For instance, a living thing (Type:*/people/person*) is more credible than a non-living thing (Type: */book/written_work*) when they suppose to be filled in the incomplete triple (*?, was_born_in, the State of Hawaii*). To put it another way, although both triples (*Donald Trump, was_born_in, the State of Hawaii*) and (*Pride and Prejudice, was_born_in, the State of Hawaii*) are not true, but we still believe that the type evidential trustiness of the former one is higher than the trustiness of the latter due to their distinct types, i.e., the type of *Donald Trump* (*/people/person*) is more reasonable for it.

**Figure 3:**
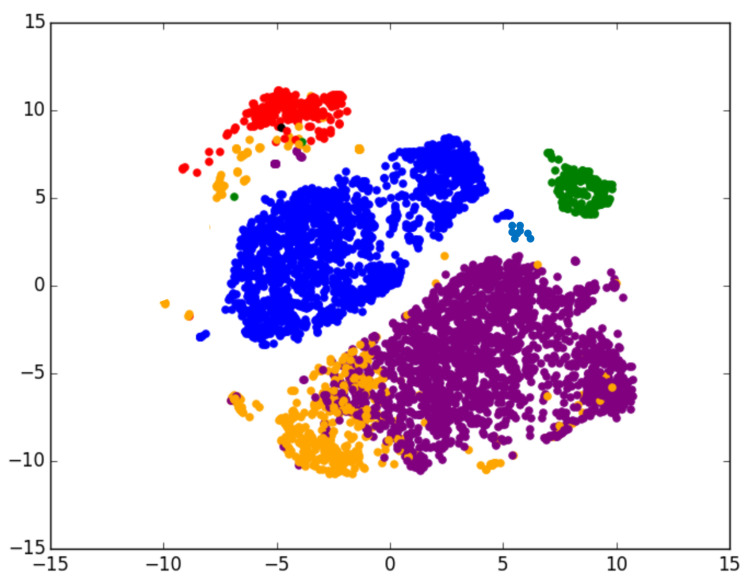
A plot of entities with entity types (Red:/education/educational_institution, Blue:*/film/film*, Purple: */tv/tv_actor*, Orange:/book/author, Green:/tv/tv_program, Black:/music/instrument). Entities with the same entity type tend to appear in well-defined clusters in the embedding space.

**Entity Type Triple.** The key motivation is based on the observation in the research of KG embedding that the learned entity embeddings can be clustered well according to their entity types in the embedding space [34]. For instance, Figure 3 shows that the entity embeddings cluster well according to their entity types represented by different colors [49]. The blue dots indicate the entities with the type: */film/film* and the film entities appear close to each other in the embedding space. Moreover, the more similar between entity types, the more close between corresponding entities in the space, and vice versa. For instance, the group of entities with types: */tv/tv_actor* and */book/author* are closer to each other than entities with other types, and they even show some overlap. These entities share some common types including */person/person*, which is the reason that they are close to each other in the embedding space. Therefore, we believe that one of the premises of a triple (*head entity, relationship, tail entity*) holds is that the corresponding entity types first conform to this relationship. Hence, we build the entity type triple: (*head type, relationship, tail type*) by replacing both head entity and tail entity with their corresponding hierarchical types: (h,r,t)→$\left(\tau_{h},r,\tau_{t}\right)$.

**Entity Type Embedding.** We encode the entity hierarchical type information into representation learning with a general form. Suppose an entity *e* has hierarchical type: $/\tau_{e}^{\left(1\right)}/\tau_{e}^{\left(2\right)}/\ldots /\tau_{e}^{\left(m\right)}$, *m* is the number of layers in the hierarchical structure, we utilize the weighted hierarchical embedding (WHE) method, considering that different granularities of sub-type in hierarchical structures may vary in significance in type representation, to build the entity type representation $\tau_{e}$ as follows:

(3) $$\tau_{e}=\sum_{i=1}^{m}\beta_{i}\cdot \tau_{e}^{\left(i\right)}=\beta_{1}\cdot \tau_{e}^{\left(1\right)}+\cdots +\beta_{m}\cdot \tau_{e}^{\left(m\right)},$$

in which $\tau_{e}^{\left(i\right)}$ is the representation of *i*-th sub-type $\tau_{e}^{\left(i\right)}$, $\beta_{i}$ is the corresponding weight of $\tau_{e}^{\left(i\right)}$.

**Entity Type Trustiness (TT).** As mentioned above, we have entity type triple $\left(\tau_{h},r,\tau_{t}\right)$ by replacing entity with entity type. We assume that the more a type triple fits the translation assumption, the more convincing the corresponding entity triple should be considered. Hence, the distance G(·) of entity type triple $\left(\tau_{h},r,\tau_{t}\right)$ under translation-framework with entity type embedding (calculated by (3)), as follows:

(4) $$G\left(\tau_{h},r,\tau_{t}\right)={∥\sum_{i=1}^{m_{1}}\beta_{i}\cdot \tau_{h}^{\left(i\right)}+r-\sum_{j=1}^{m_{2}}\beta_{j}\cdot \tau_{t}^{\left(j\right)}∥}_{2}^{2}\;,$$

where $m_{1}$ and $m_{2}$ denote the number of layers in the hierarchical type structure of head entity and tail entity respectively. $\tau_{h}^{\left(i\right)}$ is the representation of *i*-th sub-type $\tau_{h}^{\left(i\right)}$, $\tau_{t}^{\left(j\right)}$ is the representation of *j*-th sub-type $\tau_{t}^{\left(j\right)}$, $\beta_{i}$ and $\beta_{j}$ are the corresponding weight of $\tau_{h}^{\left(i\right)}$ and $\tau_{t}^{\left(j\right)}$ respectively.

To measure the entity type trustiness during training, we first judge the current conformity of each entity type triple with translation assumption. Following margin-based training strategy, we design a function to estimate the type triple quality $Q_{\tau }\left(\tau_{h},r,\tau_{t}\right)$ as follows:

(5) $$Q_{\tau }\left(\tau_{h},r,\tau_{t}\right)=-\left(\gamma_{\tau }+G\left(\tau_{h},r,\tau_{t}\right)-G\left(\tau_{h}^{\prime },r,\tau_{t}^{\prime }\right)\right),$$

where $\gamma_{\tau }>0$ is a hyperparameter. $\left(\tau_{h}^{\prime },r,\tau_{t}^{\prime }\right)$ is a negative entity type triple in which the head type or tail type is replaced by a random one. A higher $Q_{\tau }\left(\tau_{h},r,\tau_{t}\right)$ value indicates a better entity type triple judged by the translation framework. All entity type triples are supposed to be correct at the beginning of learning, and set the entity type trustiness TT(h,r,t)=1 for all triples. Since the embeddings of both entity type and relation will be updated constantly in the learning process, the current entity type trustiness for each triple should change according to how much this entity type triple comply with the translation framework. Hence, we utilize the strategy for updating the entity type trustiness TT(h,r,t) according to its type triple quality $Q_{\tau }\left(\tau_{h},r,\tau_{t}\right)$ as follows:

(6) $$\begin{array}{c} TT\left(h,r,t\right)=\left\{\begin{array}{cc} \mu \cdot TT\left(h,r,t\right), & Q_{\tau }\left(\tau_{h},r,\tau_{t}\right)\leq 0\\ \min\{TT\left(h,r,t\right)+\nu ,1\}, & Q_{\tau }\left(\tau_{h},r,\tau_{t}\right)>0\;, \end{array}\right. \end{array}$$

where μ∈(0,1) and ν>0 are hyper-parameters, TT(h,r,t)∈(0,1]. The condition $Q_{\tau }\left(\tau_{h},r,\tau_{t}\right)\leq 0$ indicates that the current entity type triple doesn’t fit the translation rule well, and thus should cut down the corresponding entity type trustiness, otherwise should increase it when $Q_{\tau }\left(\tau_{h},r,\tau_{t}\right)>0$ holds. Hence, a higher TT(h,r,t) implies that the triple is more convinced to hold according to entity type constraints.

#### 3.3.2. Triple Trustiness with Entity Descriptions

In the following, we introduce a novel approach to build triple trustiness with entity descriptions.

**Entity Description Triple.** TT would fail to work if the types of head and tail exactly match but the fact is actually false, such as *(Donald Trump, was_born_in, the State of Hawaii)*. However, the entity textual descriptions can discover semantic relevance and offer precise semantic expression [41]. The semantic relevance between entities is capable to recognize the true triples, and precise semantic expression could promote the discriminative ability between two triples. Here, we design entity description triple to estimate the triple trustiness by replacing both head and tail with their corresponding descriptions: (h,r,t)→$\left(d_{h},r,d_{t}\right)$.

**Entity Description Embedding.** From each short description, we generate a set of keywords, which is capable of capturing the main ideas of entities, based on TFIDF. The assumption is that similar entities should have similar descriptions, and correspondingly have similar keywords. Those triple trustiness may be detected in the internal contact of their keywords. We formulate entity descriptions as $d_{e}:=\left\{w_{1},w_{2},\ldots ,w_{n}\right\}$. $\left\{w_{1},w_{2},\ldots ,w_{n}\right\}$ is the set of keywords in entity description. *n* is the size of words set. We take advantage of convolutional neural network (CNN) [43,50] to model entity description $d_{e}$. The CNN model can take word orders, i.e., complicated local interactions of keywords in entity description, into consideration. Specifically, the *i*-th output vector of convolution layer in CNN is calculated as:

(7) $$z_{i}^{\left(\ell \right)}=\sigma \left(W^{\left(\ell \right)}\cdot {w_{i}^{\prime }}^{\left(\ell \right)}+b_{i}^{\left(\ell \right)}\right)\;,$$

where $W^{\left(\ell \right)}$ is the convolution kernel for all input vectors of *ℓ*-th convolution layer after window process and $b_{i}^{\left(l\right)}$ is the optional bias. σ is the activation function such as *tanh* or *ReLU*. ${w_{i}^{\prime }}^{\left(\ell \right)}$ is the *i*-th vector of ${w^{\prime }}^{\left(\ell \right)}$ which is obtained by concatenating κ column vectors in *i*-th window of the polling output of (*ℓ*-1)-th layer. The pooling process shrinks the parameter space of CNN and filter noises after every convolution layer. We use *n*-max-pooling and mean-pooling strategies respectively in different pooling layers. After the last pooling layer, we obtain the representation of entity description $d_{e}$.

**Entity Description Trustiness (DT).** Under translation-assumption, we build the distance H(·) of the entity description triple $\left(d_{h},r,d_{t}\right)$:

(8) $$H\left(d_{h},r,d_{t}\right)={∥d_{h}+r-d_{t}∥}_{2}^{2}\;,$$

where $d_{h}$ and $d_{t}$ stand for the representation of head descriptions and tail descriptions respectively which are calculated by CNN. To measure the entity description trustiness during training, like the approach in (5), we design a function to estimate the description triple quality $Q_{d}\left(d_{h},r,d_{t}\right)$ as follows:

(9) $$Q_{d}\left(d_{h},r,d_{t}\right)=-\left(\gamma_{d}+H\left(d_{h},r,d_{t}\right)-H\left(d_{h}^{\prime },r,d_{t}^{\prime }\right)\right),$$

where $\gamma_{d}>0$ is a hyperparameter. $\left(d_{h}^{\prime },r,d_{t}^{\prime }\right)$ is a negative entity description triple in which the head description or tail description is replaced by a random one. Formally, the entity description trustiness DT(h,r,t) changes with its description triple quality $Q_{d}\left(d_{h},r,d_{t}\right)$ as follows:

(10) $$\begin{array}{c} DT\left(h,r,t\right)=\left\{\begin{array}{cc} u\cdot DT\left(h,r,t\right), & Q_{d}\left(d_{h},r,d_{t}\right)\leq 0\\ \min\{DT\left(h,r,t\right)+\nu ,1\}, & Q_{d}\left(d_{h},r,d_{t}\right)>0\;, \end{array}\right. \end{array}$$

where DT(h,r,t)∈(0,1]. A higher DT implies that the triple is more probable to hold according to entity semantic relevance learned by entity descriptions.

#### 3.3.3. Overall Triple Trustiness Model

Here we introduce the overall triple trustiness. Specifically, the overall triple trustiness model combines with two kinds of trustiness stated above: (1) entity types trustiness TT(h,r,t); (2) entity descriptions trustiness DT(h,r,t). Hence, we have overall triple trustiness model T(h,r,t) as follows:

(11) T(h,r,t)=λ·TT(h,r,t)+(1−λ)·DT(h,r,t),

where λ∈(0,1) are hyper-parameters.

## 4. Cross Entropy Loss Function for Optimization

Cross entropy is an important measurement approach of information entropy (IE) (originally proposed by Shannon in [51]). For training the model parameters, we minimize the following binary cross entropy loss function in this work:

$$\begin{array}{c} L\left(\Theta \right)=\left\{-\sum_{(h,r,t)\in D}\log p(h,r,t)-\sum_{\left(h^{\prime },r,t^{\prime }\right)\in D^{\prime }}\log(1-p\left(h^{\prime },r,t^{\prime }\right))\right\}\cdot T\left(h,r,t\right), \end{array}$$

in which we apply the logistic sigmoid function σ(·) to the model scores, that is p(h,r,t)=σ(−M(h,r,t)). Θ are all the parameters of our model including the embeddings of all entities, relations, sub-types, and keywords, i.e., Θ={E,R,T,W}, initialized randomly. (h,r,t) are the observed triple fact in the training set D and $\left(h^{\prime },r,t^{\prime }\right)$ are the negative one, the head or tail of which is replaced by a random one. Note that we do not replace both head and tail with random one at the same time. A triple will not be considered as a negative example if it is already in training set D. Here the triple trustiness T(h,r,t) are determined by (11), which instructs our model to pay more attention on those more convincing facts. $D^{\prime }$ represents the negative triple set.

$$\begin{array}{cc} D^{\prime }:=\left\{\left(h^{\prime },r,t\right)|\left(h,r,t\right)\in D\cap h^{\prime }\in E\cap h^{\prime }\neq h\right\}\\ \cup \{\left(h,r,t^{\prime }\right)|\left(h,r,t\right)\in D\cap t^{\prime }\in E\cap t^{\prime }\neq t\} & \;. \end{array}$$

It is not absolutely necessary to use a entropy loss function [34]. However, it is very common to use entropy loss for learning embeddings (like ConvE [39], FRN [52], etc) just as our model did.

**Optimization.** We use mini-batch stochastic gradient descent (SGD) for optimization. We perform the following procedure iteratively for a given number of iterations. First, we sample a small set (minibatch) of triples from the training set D, and then for each positive triple in it, we construct a negative sample by replacing the head or tail with a random one. The parameters are then updated by taking a gradient descent step gradually. Algorithm 1 shows the optimization algorithm in detail. As pointed out by [53,54], it would be uneconomical to save all negative properties of an entity or a concept. Therefore, we further require entities to have non-negative vectorial representations. In fact, the distributed representations can be taken as the feature vectors for entities, with latent semantics encoded in different dimensions. To better compare different entities on the same scale, we further require entity representation to stay within the hypercube of ${\left[0,1\right]}^{\kappa }$, as approximately Boolean embeddings. In most cases, non-negative will further induce sparsity and interpretability.

**Algorithm 1** Learning TransT using cross entropy loss function.
**Require:** Training set $D=\left\{\left(h,r,t\right)\right\}$, the set of entity types, entity descriptions.**Ensure:** The embeddings of all entities, relations, sub-types, and keywords: Θ={E,R,T,W}.1:
**Initialize**
2:e←Gaussian(0,1)/10 for each $e\in E,e\in R^{\kappa }$3:r←Gaussian(0,1)/10 for each $r\in R,r\in R^{\kappa }$4:t←Gaussian(0,1)/10 for each $t\in T,t\in R^{\kappa }$5:w←Gaussian(0,1)/10 for each $w\in W,w\in R^{\kappa }$6:
**Loop**
7:$\;D_{batch}\leftarrow sample\left(D,m\right)$//minibatch size *m*8:$\;A_{batch}\in \phi$//initialize training set as null9:
for $\left(h,r,t\right)\in D_{batch}$ do
10:$\;\;(h^{\prime },r,t^{\prime })\leftarrow sample\;D$ //corrupted11:
$\;\;A_{batch}\leftarrow A_{batch}\cup \left(\left(h,r,t\right),\left(h^{\prime },r,t^{\prime }\right)\right)$
12:
endfor
13:  Update embeddings w.r.t.14:    $\sum_{A_{batch}}\nabla [-\log p\left(h,r,t\right)-\log\left(1-p\left(h^{\prime },r,t^{\prime }\right)\right)]\cdot T\left(h,r,t\right)$15:
**End Loop**



## 5. Experiments

We present three experiments: KG noise detection, KG completion and triple classification to demonstrate the effectiveness of our proposed model. We first introduce the datasets, experimental settings, and baselines for comparison, and then show the experimental results and discussions.

### 5.1. Datasets

Our experiments are conducted on three public benchmark datasets FB15K-N1, FB15K-N2, and FB15K-N3 (The datasets can be accessed at https://github.com/thunlp/CKRL) which are generated based on FB15K with different noise rates (i.e., 10%, 20%, and 40% respectively) to simulate the real-world KG construction with errors [23]. FB15K [17] is a typical experimental dataset extracted from Freebase. FB15K contains 14951 entities and 1345 relationships, in which all entities possess descriptions. Moreover, we collect 3851 entity types from FB15kET (The FB15kET can be accessed at https://github.ncsu.edu/cmoon2/kg) [49].

Given a positive triple (h,r,t) in KG, the head or tail is randomly replaced to form a negative one $\left(h^{\prime },r,t\right)$ or $\left(h,r,t^{\prime }\right)$. In order to generate harder and more confusing noises, $h^{\prime }$ (or $t^{\prime }$) should have appeared in the head (or tail) position with the same relation, which means that the tail entity of relation *was_born_of* in negative triples should also be a place. All three noisy datasets share the same entities, relations, validation and test sets with FB15K, and all generated negative triples fused into the original training set of FB15K. The statistics are listed in Table 1 and Table 2.

### 5.2. Experimental Settings and Baselines

In the experiment, we evaluate our TransT model with two different combination strategies. TransT (TT) considers entity type trustiness, while TransT(TT+DT) considers both entity type trustiness and entity description trustiness. We choose two models as the baselines for comparison: (1) TransE which is a typical model used for entity prediction [17], and (2) CKRL which is a state-of-the-art model focusing on representation learning on noisy knowledge graph [23]. The results for the baselines are directly taken from original literature. We train our TransT model using mini-batch SGD. We select the learning rate in the stochastic gradient descent among {0.0001, 0.001, 0.01}, the dimension of entity, relation, entity type, and keyword embedding κ in all models in a range of {50, 100} on the validation set. For overall triple trustiness model, the hyperparameter λ set as 0.5, $\gamma_{\tau }=\gamma_{d}=1$, μ=0.95,ν=0.05. For CNN, we set the parameters are: #window size=2, #convolution layer = 2, #dimension of feature map = κ. Usually, m=2 and set $\beta_{1}=\beta_{2}=1/m$ in FB15K.

### 5.3. Kg Noise Detection

To verify the capability of our TransT models in identifying noises in KGs, we conduct a comparative experiment – KG noise detection according to their triple scores.

**Evaluation Protocol.** We utilize translation-assumption method TransE: M(h,r,t)=${∥h+r-t∥}_{2}^{2}$ as our triple model. Following the triple classification protocol in [19], we rank all triples in training set with their model score. Therefore, the higher the model score, the more likely the triple is noise. We use precision/recall curves to show the performances.

**Figure 4:**
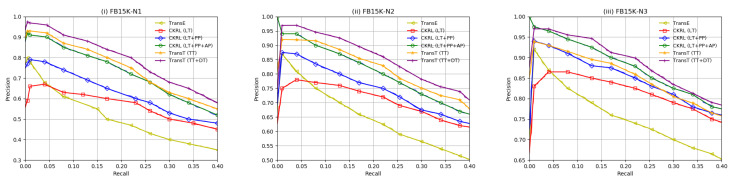
**KG noise detection results.** Evaluation on FB15K-N1, FB15K-N2, and FB15K-N3.

**Experimental Results.**Figure 4 demonstrates the evaluation results of KG noise detection, from which we can observe that: (1) Our proposed trustiness-aware model TransTs broadly achieves the best performances on all three datasets with different noise rates, which confirms the capability of our TransT models in modeling tripe trustiness and detecting errors in knowledge graphs. (2) TransT (TT+DT) has an impressive improvement in error detection compared to TransT (TT). It indicates that the triple trustiness with entity descriptions can provide significant help for error detection. (3) In addition, TransT (TT+DT) has 60∼78% in precision with different noise rates when the recall approximately equals to 40%, which demonstrates the triple trustiness strategy could help for noisy KG embedding. (4) With the noises level rising, TransT (TT+DT) performs better regarding to noise detection. We believe the main reason is that the triples in FB15k-N3 has lower confidence than in FB15k-N1, considering the datasets as bipartite graphs. For instance, there are 671,067 training triples and 187,925 noises in FB15k-N3, while FB15k-N1 only has 529,550 triples and 46,408 noisy triples. Due to higher noise rate, the knowledge in FB15k-N3 can be more easily disturbed by noisy data, which can be significantly detected by our models.

### 5.4. Kg Completion

The classical KG completion task concentrates to complete a triple when one of its head, relationship or tail is missing, i.e., to predict how likely some additional triples are held, which aims to verify the capability of our proposed model for KG completion.

**Evaluation Protocol.** We conduct entity prediction determined by TransE [17]: h+r≈t. We use the ranking criteria for evaluation. Firstly for each test triple, we remove the head entity and replace it by each of the entities of the dictionary in turn. The function value $M(h^{\prime },r,t)$ of the negative triples would be computed by the related models and then sorted by descending order. We can obtain the exact rank of the correct entity in the candidates. Similarly, we repeat the whole procedure while removing the head entity instead of the tail entity of the test triple. Finally, we use two evaluation metrics for comparison: the mean of those predicted ranks (Mean Rank) and the proportion of correct entities ranked in the top 10 (Hits@10(%)). We also follow the different evaluation settings of “Raw” and “Filter” utilized in [17].

**Experimental Results.**Table 3 shows the results of entity prediction with different noise rates, from which we observe that: (1) All TransT models achieve better performance compared with the baseline on all noisy datasets, which confirms the capability of our models in KG completion beyond KG noise detection. (2) Our methods achieve more significant improvement as the noise rate increases, compared with basic mode TransE between the three noisy datasets. It verifies that considering the trustiness in noisy KG embedding is very essential especially when KGs have a high rate of noises. Specifically, according to the metrics Mean Rank (Filter) and Hits10(%) (Filter), TrustT (TT+DT) improves (7, 2.5%), (13,3.8%) and (23, 5.3%) on FB15kET-N1, FB15kET-N2, and FB15kET-N3 respectively. (3) TransT (TT+DT) perform better than TransT (TT). It demonstrates that the entity description information could further benefit KG completion especially when TT fails.

### 5.5. Triple Classification

Triple classification aims to judge whether a triple in test data holds or not, which could be viewed as a binary classification problem, and also can be regarded as a noise detection task in test data.

**Evaluation Protocol.** Since there are no explicit negative triples in existing KGs, we build negative triples in validation and test set with an equal number of positive and negative examples. Following the same protocol in [19], we use the validation set to find a threshold ζ. If the model score ||h+r−t||≤ζ in classification, the triple will be classified to be true, otherwise to be false. The final accuracy is based on how many triples are classified correctly.

**Experimental Results.**Table 4 shows the accuracy of the evaluation result of different models. We can find that: (1) The TransT models perform better than the baseline on three datasets, and the improvements become more larger with higher noise rates, which prove that triple trustiness can be helpful for relation triple classification as well. (2) Specifically, TransT (TT+DT) model improves 0.7%, 0.9% and 1.8% on FB15K-N1, FB15K-N2, and FB15K-N3 respectively, it reaffirms that our method becomes more significant with higher noise rates. (3) However, the traditional model TransE may also achieve comparable results, and the improvement our proposed model has over them in this task seems to be unobvious. It may be because our proposed models mainly focus on calculating trustiness for triples in training set, but not for negative triples that are generated in the testing set.

## 6. Conclusions and Future Work

In this paper, we concentrate on noisy knowledge graph embedding with triple trustiness. We consider to estimate the triple trustiness according to the conventional external nonstructural auxiliary information, i.e., entity type instances and entity descriptions. Correspondingly, we propose two sub-models for calculating triple trustiness with entity types and entity descriptions respectively. Through extensive experiments on three real-world datasets, we demonstrate TransT’s effectiveness over the baselines. In the future, we will explore the following directions: (1) More external resources can further improve our model. We will explore to combine more external heterogeneous information with internal structural information to further enhance the performance. (2) Network embedding also faces the noise issue. We will apply our proposed framework to improve network embedding as well. (3) Graph Signal Processing (GSP) [55,56], which aims to generalize the classical signal processing to graph signals, could also benefit from KG embedding approaches as this work proposed.

## Figures and Tables

**Table 1:** Statistics of FB15K and FB15kET. FB15kET provides entity type information: (<entity, entity type>). We only use the training data of FB15kET, not valid and test data, to estimate type trustiness.

**Dataset**	**#Entities**	**#Rel**	**#Train**	**#Valid**	**#Test**
FB15k	14,951	1345	483,142	50,000	59,071
**Dataset**	**#Ent**	**#Type**	**#Train**	**#Valid**	**#Test**
FB15kET	14,951	3851	136,618	16,000	16,000

**Table 2:** Statistics of the FB15k-N1, FB15K-N2, FB15K-N3 used for experiments. #Negative triples denotes the number of noises in them.

Datasets	FB15k-N1	FB15k-N2	FB15k-N3
#Negativetriples	46,408	93,782	187,925
#Trainingtriples	529,550	576,924	671,067
#Validtriples	50,000	50,000	50,000
#Testingtriples	59,071	59,071	59,071

**Table 3:** **Entity prediction results.** Evaluation of different models on FB15K-N1, FB15K-N2, and FB15K-N3.

Dataset	FB15K-N1				FB15K-N2				FB15K-N3			
Metrics	Mean Rank		Hits@10(%)		Mean Rank		Hits@10(%)		Mean Rank		Hits@10(%)	
	Raw	Filter	Raw	Filter	Raw	Filter	Raw	Filter	Raw	Filter	Raw	Filter
TransE	240	144	44.9	59.8	250	155	42.8	56.3	265	171	40.2	51.8
CKRL (LT)	237	140	45.5	61.8	243	146	44.3	59.3	244	148	42.7	56.9
CKRL (LT+PP)	236	139	45.3	61.6	241	144	44.2	59.4	245	149	42.8	56.8
CKRL (LT+PP+AP)	236	138	45.3	61.6	240	144	44.2	59.3	245	150	42.8	56.6
TransT (TT)	233	**137**	45.8	61.2	239	143	44.6	58.1	249	153	42.4	55.2
TransT (TT+DT)	**232**	**137**	**45.9**	**62.3**	**237**	**141**	**45.0**	**60.1**	**246**	**148**	**43.4**	**57.1**

**Table 4:** **Triple classification results.** Evaluation of different models on FB15K-N1, FB15KN2 and FB15K-N3.

Dataset	FB15K-N1	FB15K-N2	FB15K-N3
TransE	81.3	79.4	76.9
CKRL(LT)	81.8	80.2	78.3
CKRL(LT+PP)	81.9	80.1	78.4
CKRL(LT+PP+AP)	81.7	80.2	78.3
TransT (TT)	82.2	80.8	79.1
TransT (TT+DT)	**82.4**	**81.1**	**80.1**
